# Improving the size selectivity and exploitation pattern of cocktail shrimp (*Trachypenaeus curvirostris*) in shrimp trawl fishery of the South China Sea

**DOI:** 10.1371/journal.pone.0295776

**Published:** 2023-12-13

**Authors:** Bingzhong Yang, Bent Herrmann, Rong Wan

**Affiliations:** 1 Key Laboratory of Open-Sea Fishery Development, Ministry of Agriculture and Rural Affairs, South China Sea Fisheries Research Institute, Chinese Academy of Fishery Sciences, Guangzhou, 510300, China; 2 College of Marine Science, Shanghai Ocean University, Shanghai, 201306, China; 3 SINTEF Ocean, Fishing Gear Technology, Hirtshals, Denmark; 4 University of Tromsø, Breivika, Tromsø, Norway; 5 DTU Aqua, Technical University of Denmark, Hirtshals, Denmark; Hellenic Center for Marine Research, GREECE

## Abstract

In order to improve the size selectivity and exploitation pattern for cocktail shrimp (*Trachypenaeus curvirostris*) in shrimp trawl fishery of the South China Sea (SCS), selective properties of four codends were tested and compared. These experimental codends involved two mesh sizes, 30 and 35 mm, and two mesh shapes, diamond-mesh (T0) and diamond-mesh turned by 90 degree (T90), respectively. Our results demonstrated that increasing the mesh sizes in T0 codends or/and applying T90 codends would improve the selective properties for cocktail shrimp in the SCS. By comparing selectivity parameters, delta selectivity and exploitation pattern indicators, the T90 codend with 35-mm mesh size (T90_35) presented the best selective properties for cocktail shrimp in the studied areas. It will be a potential choice to substitute the currently legal codend in fisheries management to mitigate the bycatch of undersized cocktail shrimp in shrimp trawl fisheries of the SCS.

## Introduction

Cocktail shrimp (*Trachypenaeus curvirostris*), also named as ‘Ying-zhua-xia’ in Chinese, is an social-economically relevant species in the shallow waters of Indo-West Pacific, widely distributed from China and Australia to East Africa and the Red Sea [1,2]. In China, cocktail shrimp has always been a major shrimp species with its annual landing recorded in the official documentation, China’s Fisheries Statistical Yearbooks. Based on these figures, however, the catch volume of cocktail shrimp declined from 312,436 t in 2000 to 239,243t in 2021, presenting a decrease of 23.43% [3,4]. Additionally, the catch efficiency of cocktail shrimp was recorded to decline from 180.9 kg/m^2^ in the 1980s to 16.8 kg/m^2^ in the 1990s by Song et al. [5] in the East China Sea.

Similar to many finfish and shrimp species in the South China Sea (SCS), the declined and overexploited state of cocktail shrimp stock could be attributed to a lot of factors [6], among them the fishing gears and related management regulations are of concern. Due to its benthic-distributed characteristics, demersal trawls are the main gears to target cocktail shrimp in China [7,8]. To retain a small-size species like cocktail shrimp, small mesh sizes in the codends are required in the demersal trawls. At present, the minimum mesh size (MMS) in the codends for all trawls targeting shrimp species is regulated as 25 mm in China. This MMS regulation, however, has been questioned in several studies [9–11].

Specifically, the codend with a MMS of 25 mm (D25) has been tested to have serious bycatch issues. For instance, Yang and Herrmann [8] recently tested the size selectivity and exploitation patterns of six diamond-mesh codends, with identical netting characteristics but mesh sizes ranging from 25 to 54 mm, for cocktail shrimp in demersal trawl fisheries of the SCS. Their results demonstrated that the size selectivity of the legal D25 was poor and increasing the mesh sizes had little effect on improving the size selectivity and exploitation pattern for the target species in the studied areas. Similar to other studies of diamond-mesh codends for different species, the poor and variable size selectivity of diamond-mesh codends could be explained by the mechanical characteristics of the netting used and its working principle. During fishing, the diamond-mesh codends would be distorted into a bulbous shape with the accumulation of the catch and towing force from the vessels [12–14]. In that situation, meshes in the forward section of the codends tended to be closed, while only meshes just ahead of the catch bulk would stay open and available for target species to escape [15,16]. One simple option to circumvent this drawback of diamond-mesh codends is just turning the netting direction by 90°, changing to T90 codends [14,17,18]. Compared with the traditional diamond-mesh codends (T0), T90 codends would keep the meshes more open due to that the wider axis of the knots are oriented perpendicular to the towing force [19]. Therefore, T90 codends have great potential to improve the size selectivity for cocktail shrimp. However, the size selectivity and exploitation pattern of T90 codends have never been tested for this species in the SCS.

In order to address the by-catch issue of undersized individuals for cocktail shrimp trawl fisheries, we tested and compared four codends with different mesh shapes, T0 and T90, and mesh sizes, 30 and 35 mm, in the SCS. We focused on the following research questions: 1) how would the size selectivity and exploitation pattern of different codends for cocktail shrimp change with the mesh shapes or/and mesh sizes changed, and 2) if these potential changes were length-dependent or not.

## Materials and methods

### Ethics statement

This study did not involve any endangered or protected species. Experimental fishing was conducted onboard a commercial vessel in accordance with the fishing permit granted by the Ministry of Agriculture and Rural Affairs of China. No other authorization or ethics board approval was required to carry out this study. Information on animal welfare and steps to ameliorate suffering and methods of sacrifice is not applicable, as the animals were not exposed to any additional stress other than that involved in commercial fishing practices.

### Sea trials and experimental setup

Sea trials were conducted onboard a commercial shrimp trawler ‘Gui-bei-yu 96899’ (38 m, 280 kW) in the Southern China Sea, around the Weizhou Island, which is a traditional fishing ground for commercial shrimp trawling, in November 2020. We selected this area due to that a previous codend selectivity experiment was finished by Yang and Herrmann [8] and they got sufficient number of individuals for the species investigated. We expected that this area would have a potential for us to answer the research questions and make comparison with the previous study.

**Fig 1:**
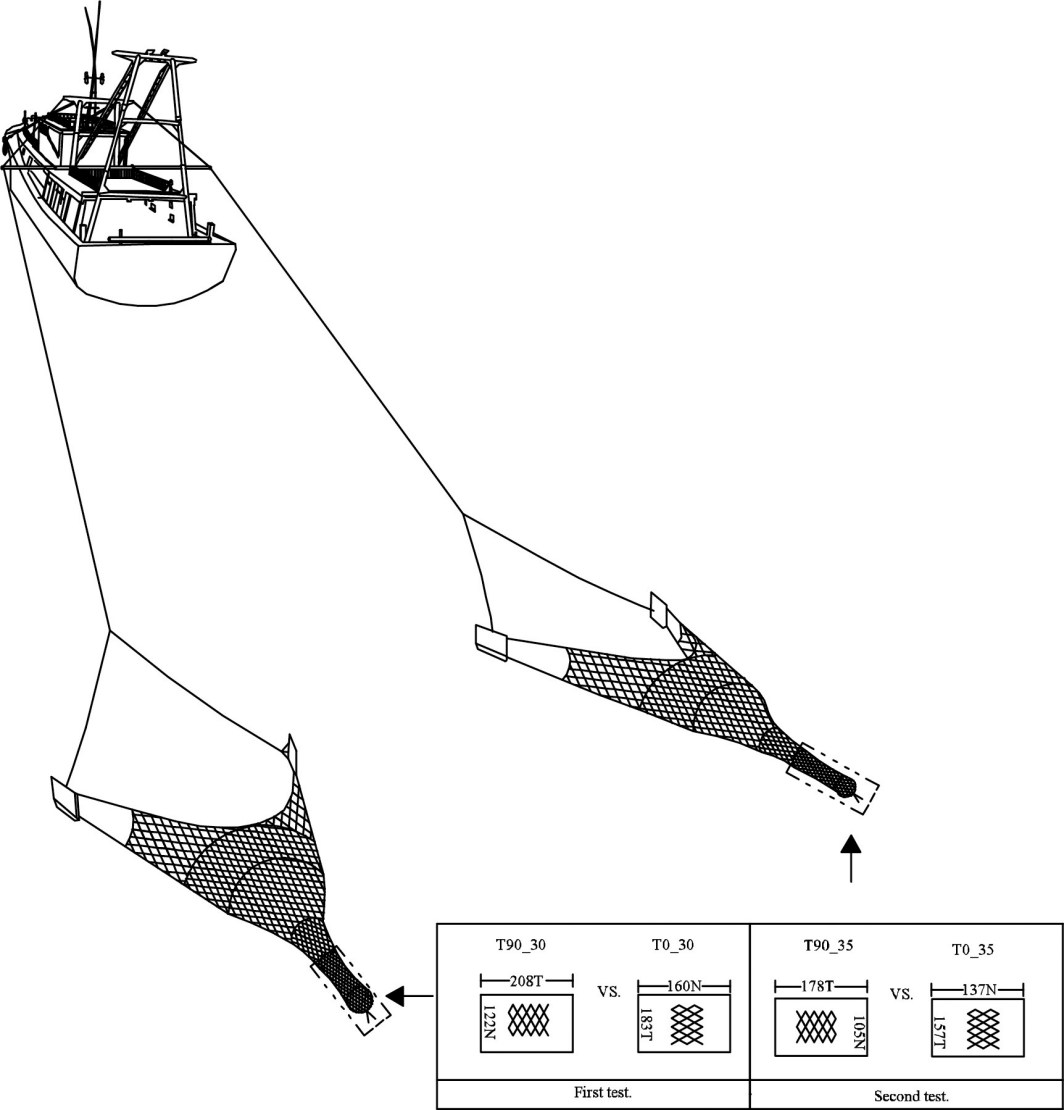
Schematic view of the trawling system and the experimental codends.

We used the trawl-net components of the commercial fishing vessel except the codends, which were the gear modifications we would like to test. The selected vessel operates a double-rigged system with outrigger booms from where two identical trawls are towed with a set of similar-size otter boards each (Fig 1). The trawl-nets used were two-panel structure, made of 45-mm polyethylene (PE) meshes with a circumference of 860 meshes at the entrance of the belly. The footrope length was 36-m long and the length of the headline was about 28 m. To maintain the trawl in contact with the seabed, an 80 m long chain (weighted about 210 kg) was lashed to the footrope to act as ground gears. Two sets of flat wooden otter boards, weighted 250 kg, were applied to spread the trawls in horizontal direction. The wing-end of the trawls was connected to the otter boards using bridles about 0.5 m in length (Fig 1). During commercial fishing, the otter board spread was about 15 m while the vertical height of the headline was normally 1.5 m.

Four experimental codends, with different mesh sizes and mesh shapes, were designed based on designs by Yang and Herrmann [8]. Considering that the selective properties of the legal D25 was poor, we selected two larger mesh sizes as 30 and 35 mm, and two mesh shapes, T0 and T90, respectively. Based on the mesh shapes and sizes, these codends were termed as T0_30, T0_35, T90_30 and T90_35, respectively. For example, T0_30 was the T0 codend with a mesh size of 30 mm, while T90_30 represented the T90 codend with the same mesh size. Following the designed rule by Yang and Herrmann [8], the T0 codends had the same stretched length in circumference and longitudinal direction, while the T90 codends were 33% smaller in circumference and 30% longer in the longitudinal direction when comparing with the T0 codends with the same mesh sizes due to the hanging ratios based on past experiments [20,21]. To make sure that the T0 and T90 codends with the same mesh size had identical mesh opening, they were manufactured using the same twine netting. Specifically, the mesh opening of the T0_30 and T90_30 codend was measured to be 29.79±0.65 mm, and 35.66±1.06 mm for the T0_35 and T90_35 codend. Detailed information about the codends can be found in Fig 1.

The covered codend method [13] was applied to collect escapees of cocktail shrimp from the tested codends. Compared with the experimental codends, the cover-net was about 1.5 times larger in circumference and its mesh opening was 12 mm. Instead of using hoops, a total of 12 flexible kites were attached to the cover to remove potential masking effects [8,22,23]. To check how these kites work, we used GoPro HERO 4 to act as underwater video recording systems (see the images in supplementary material). To access easily to the catch from the codends, we mounted a zipper with a length of 1.1 m to the side of the cover-nets.

Taking full advantage of the double-rigged trawling systems in the fishing vessel, we arranged two pairwised tests as: T0_30 vs. T90_30 and T0_35 vs. T90_35 (Fig 1). Each test was planned to replicate for nine hauls. In each fishing haul, catch from the tested codends and covers were separately handled. The catch of cocktail shrimp was sorted and sub-sampled if the catch number was huge. All catch of the target species was kept in marked boxes, frozen and then length measured once got back to the laboratory on land.

### Estimation of size selectivity

The experimental setups enabled us to analyze the catch data as binomial data, due to that for a given codend tested an individual cocktail shrimp were either caught by the codend or cover. The catch probability, often expressed as *r*_*j*_*(l)* in which *j* is the ID of fishing haul and *l* is the length of the target species, can be used to model the size selectivity. However, the values of *r*_*j*_*(l)* can be varied for the same codend in different hauls or sea trials due to some uncertainties, such as hauling direction, wind speed and water depth described by Fryer (1991) [24]. In the present study, we are interested in the average size selectivity of the codends over all hauls as this provides an estimate on how the codends would perform on average in the fishery [25,26]. This average size selectivity can be described as *r*_*codend*_ (*l*, ***v***_*codend*_), in which ***v***_*codend*_ is the vector of selectivity parameters to be estimated. The estimation of these parameters can be obtained using maximum likelihood method by minimizing the following expression:

$$-\sum_{j=1}^{m}\sum_{l}\left\{\frac{nR_{lj}}{qR_{j}}\times \ln\left[r_{codend}(l,v_{codend})\right]+\frac{nE_{lj}}{qE_{j}}\times \ln\left[1-r_{codend}(l,v_{codend})\right]\right\}$$ (1)

where *nR*_*lj*_ and *nE*_*lj*_ represent the number of the target species of length class *l* in haul *j* from the codend and cover, while *qR*_*j*_ and *nE*_*j*_ represent the sub-sampled ratios for length measurement from the codend and cover, respectively.

Four models, Logit, Probit, Gompertz and Richards, were used to as candidates to fit the catch data and describe *r*_*codend*_ (*l*, ***v***_*codend*_) for each codend tested. For the first three model, two selectivity parameters, L50 (50% retention length) and SR (selectivity range, = L75-L25), were used to describe, while for the last model an additional parameter, *δ*, needs to be added [13]. The size selectivity of each codend configuration for cocktail shrimp can be estimated separately following the same procedure. First, the candidate models were all fitted to Eq 1 to calculate their Akaike’s information criterion (AIC) values [27], and select the best model, the one with the lowest AIC value. Then, applying the best model, a double-bootstrapping technique was used to obtain the Efron percentile [28] 95% confidence intervals, CIs, for the selectivity parameters and curves [8,25,26,29,30]. Finally, how the selected model fitted the experimental data could be judged based on their *p*-values. When the models were sufficiently able to describe the catch data, they would result in *p*-values >0.05. In special cases in which their *p*-values <0.05, the residuals need to be checked to see if this was due to structural problems of the models or just overdispersion in the catch data [13].

### Delta selectivity

In order to infer the potential differences in the size selectivity between different codend configurations, delta selectivity, Δ*r*(*l*), was applied using the following equation:

$$\Delta r(l)=r_{x}(l)–r_{y}(l)$$ (2)

where *r*_*x*_ (*l*) represents the size selectivity of codend *x*, and *r*_*y*_ (*l*) is the size selectivity of codend *y*. The Efron percentile 95% CIs in delta selectivity could also be calculated applying the double-bootstrapping technique mentioned above. If the CIs of delta selectivity did not cover 0.0, the difference between codends would be statistically significant.

### Estimation of exploitation pattern indicators

Besides the size selectivity and delta selectivity, it is highly relevant to measure how the codend configurations would impact the exploitation pattern for the target species in specific fishery situations. To do this, exploitation pattern indicators have been widely used by researches of fishing gear selectivity [8,31–34]. In the present study, we estimated three exploitation pattern indicators, *nP*-, *nP*+, and *dnRatio*, applying the following equations:

$$\begin{array}{c} nP-=100\times \frac{\sum_{l<MCRS}\{r_{codend}(l{,v}_{codend})\times {nPop}_{l}\}}{\sum_{l<MCRS}\{{nPop}_{l}\}}\\ nP+=100\times \frac{\sum_{l\geq MCRS}\{r_{MCRS}(l{,v}_{codend})\times {nPop}_{l}\}}{\sum_{l\geq MCRS}\{{nPop}_{l}\}}\\ dnRatio=100\times \frac{\sum_{l<MCRS}\{r_{codend}(l{,v}_{codend})\times {nPop}_{l}\}}{\sum_{l}\{r_{codend}(l{,v}_{codend})\times {nPop}_{l}\}} \end{array}$$ (3)

in which *nPop*_*l*_ is the size structure of cocktail shrimp in a specific fishing population scenario, while MCRS represent the minimum conservation reference size of cocktail shrimp (7.0 cm) in the SCS [8]. Two fishing population scenarios were created by pooling catch data, both from cover and codend, in the sea trials. One was based on the data of the present study, the other was from the previous research by Yang and Herrmann [8] which was conducted in the same fishing ground with similar trawl configuration. Again, the Efron percentile 95% CIs for the exploitation pattern indicators can be estimated through the double-bootstrapping technique.

We used the selectivity software SELNET [8,26,29,35] to conduct data analysis. To facilitate the production of plots, statistical tool R [36] and the ggplot2 package [37] were applied.

## Results

**Fig 2:**
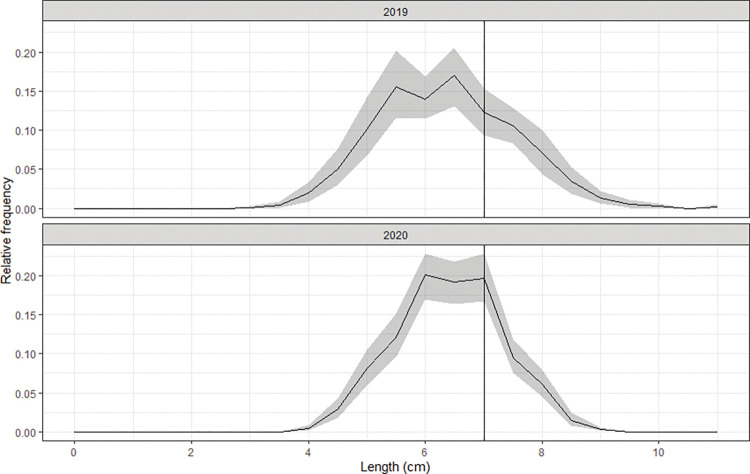
Estimated average size structures of cocktail shrimp in fishing population scenarios of 2019 and 2020. Shaded areas show the 95% confidence intervals, and the vertical lines represent the MCRS (minimum conservation reference size, 7.0 cm) of cocktail shrimp in the SCS.

During the sea trials, we conducted a total of 35 valid hauls: eight for the T0_30 codend and nine for the rest three codends. Duration of the experimental fishing was 130.74±8.72 min (ranging from 115 to 158 min), and the average water depth in the fishing grounds was 24.57±5.75 m (between 18 and 39 m). In total, 107 catch species were classified in the sea trials. Cocktail shrimp was one of the most dominant species, major bycatch species were fish, including white croaker (*Pennahia argentata*), finespot goby (*Chaeturichtys stigmatias*) and burrowing goby (*Trypauchen vagina*). Sub-sampled ratios for the target species varied between 0.25 and 1.0. In total, 2175 individuals of cocktail shrimp, of which 1382 from the tested codends and 793 from the covers, were measured and included in the size selectivity analysis. Based on the catch data from all hauls, we created a population scenario of cocktail shrimp in 2020. Additionally, the other population scenario in 2019 was generated by applying the catch data from the previous sea trials. Relative frequency in length was different in these population scenarios, in which dominant length range was 5.5 to 6.5 cm in 2019 while 6.0 to 7.0 cm in 2020, respectively (Fig 2).

### Size selectivity

By comparing the AIC values of the candidate models, the Logit model was selected for the T90_35 codend, while the Gompertz model was chosen for the rest three codends (Table 1). These selected best models all performanced well for the catch data, as they all obtained *p*-values larger than 0.05 (Table 2). The selectivity parameters showed that L50 of the T0_30 codend was significantly lower than those of the rest codends, and the value of L50 from the T90_30 codend was significantly lower than values from the T0_35 and T90_35 codend, respectively, while the T90_35 codend had higher L50 than the T0_35codend but was not statistically significant (Table 2). The value of SR from the T0_30 codend was significantly lower than those of the other three codends tested, among which no significant difference was found.

**Table 1:** Akaike’s information criterion (AIC) values from candidate models for the tested codends. Selected models in bold.

	model			
codend	Logit	Probit	Gompertz	Richards
T0_30	279.59	283.62	**275.97**	278.33
T0_35	1033.42	1036.17	**1018.51**	1020.47
T90_30	808.82	809.37	7**99.92**	802.37
T90_35	**2202.21**	2203.62	2209.72	2202.94

**Table 2:** Selectivity parameters and fit statistics obtained from the selected models for the tested codends. The selectivity parameters of D25, D30, D35, D40, D45 and D54 were from the previous study by Yang et al. [8].

	parameters					
codends	model	L50 (cm)	SR (cm)	*p*-value	deviance	DOF
T0_30	Gompertz	5.10 (4.95–5.42)	0.61 (0.44–0.76)	0.9765	2.65	9
T0_35	Gompertz	6.24 (6.10–6.41)	1.28 (0.95–1.62)	0.1087	14.4	9
T90_30	Gompertz	5.76 (5.61–5.94)	0.85 (0.70–0.99)	0.3983	9.43	9
T90_35	Logit	6.60 (6.36–6.84)	1.36 (0.93–1.78)	0.7140	7.12	10
D25		5.85 (5.16–6.18)	0.55 (0.10–0.86)			
D30		6.20 (5.37–6.44)	0.66 (0.33–1.11)			
D35		5.88 (4.26–6.35)	1.66 (0.52–4.35)			
D40		6.03 (5.73–6.83)	1.10 (0.71–1.67)			
D45		6.12 (0.10–6.82)	1.28 (0.59–4.97)			
D54		6.61 (5.67–9.30)	1.67 (1.13–3.85)			

Compared with the selectivity parameters of the previous study by Yang and Herrmann [8], the value of L50 from the T0_30 codend was lower than that of the D30 codend, and the T0_35 obtained higher L50 value than the D35 codend, but these differences were not significant (Table 2). The SR values from these codends were close to each other. It is noteworthy that the T90_35 codend from the present study had similar L50 and smaller SR to the D54 codend by Yang and Herrmann [8], though the differences were still insignificant.

**Fig 3:**
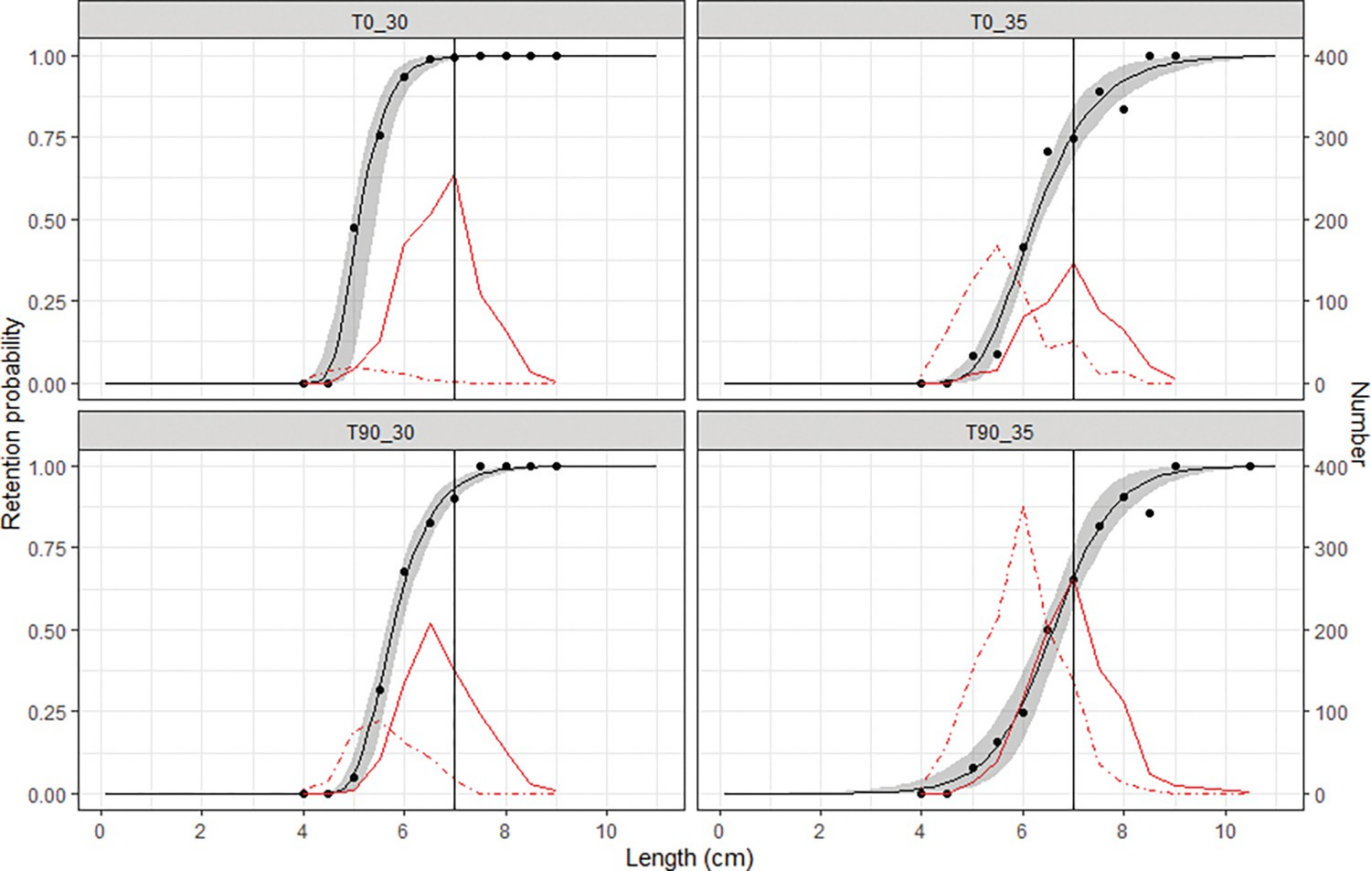
Experimental catch proportion and selectivity curves obtained for the T0 and T90 codends. Circle marks represent experimental catch proportion. Solid black curves represent selectivity curves and shaded areas describe the 95% confidence intervals. Red solid curves represent the size distribution of cocktail shrimp caught by the codends, while the red dotted curves represent the one caught by the covers. Vertical lines represent the MCRS (minimum conservation reference size, 7.0 cm) of cocktail shrimp in the SCS.

The selectivity curves showed that for an individual of the target species with a length of MCRS, the retention probability by the T0_30 codend was as high as 99.50% (CI: 98.50–99.92%), dropped to 93.23% (CI: 89.40–95.91%) and 76.00% (CI: 68.75–84.25%) for the T90_30 and T0_35 codend, respectively, while the probability further lowered to 65.79% (CI: 57.06–75.40%) for the T90_35 codend (Fig 3).

### Delta selectivity

**Fig 4:**
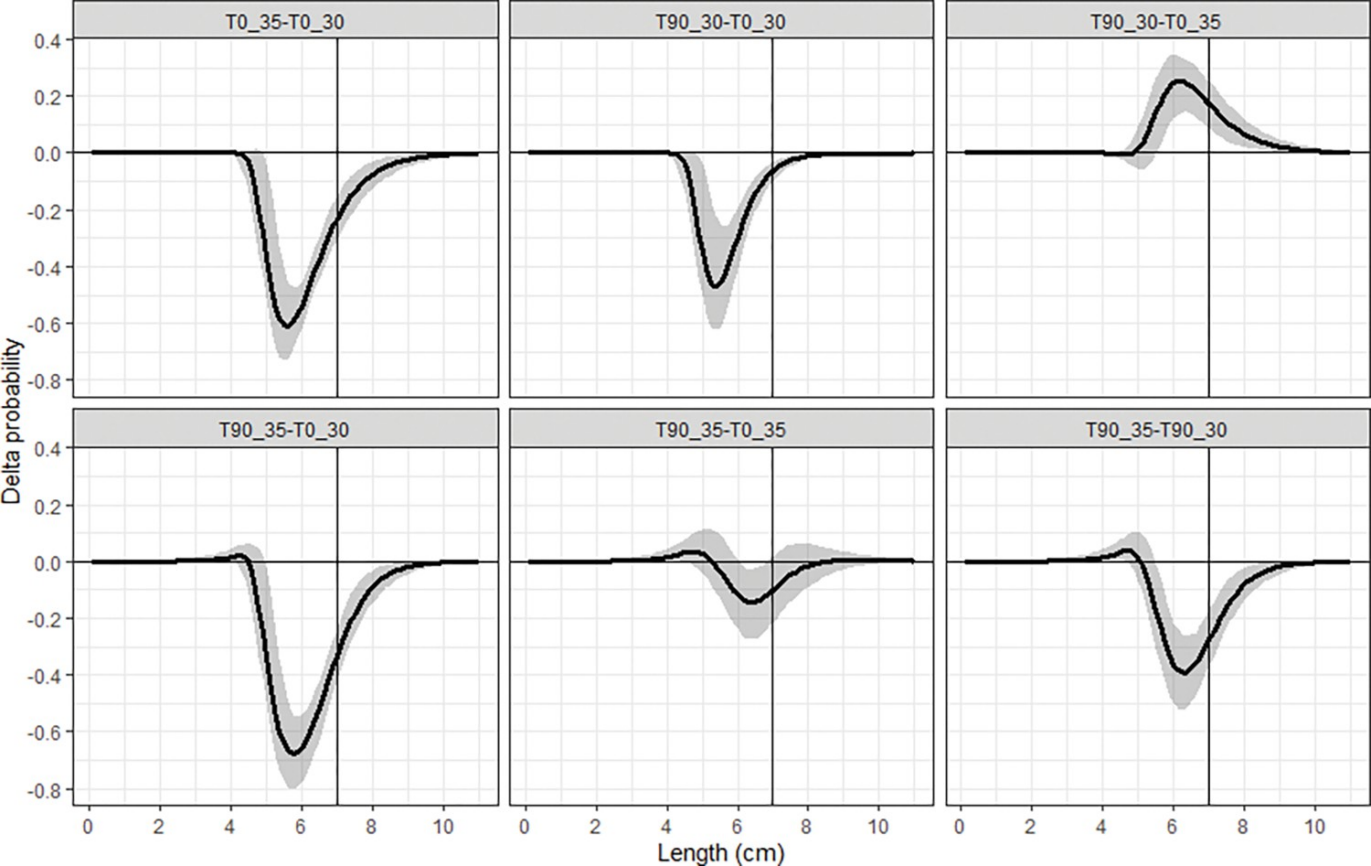
Delta selectivity of comparison between the tested codends. The black curves represent the delta selectivity and shaded areas are the 95% confidence intervals. Vertical lines are the MCRS (minimum conservation reference size, 7.0 cm) of cocktail shrimp in the SCS.

The Delta selectivity curves showed that compared to the T0_30 codend, the T0_35 and T90_30 codend would have significantly lower retention probability for cocktail shrimp with length larger than 5.0 cm, while the T90_30 codend had significantly higher retention probability for shrimp with length longer than 5.6 cm comparing the T0_35 codend (Fig 4). The T90_35 codend had significantly lower retention probability for cocktail shrimp in some length ranges than the rest codends as follow: > 5.0 cm than the T0_30 codend, >5.5 cm than the T90_30 codend, and 6.1–6.8 cm than the T0_35 codend, respectively (Fig 4).

**Fig 5:**
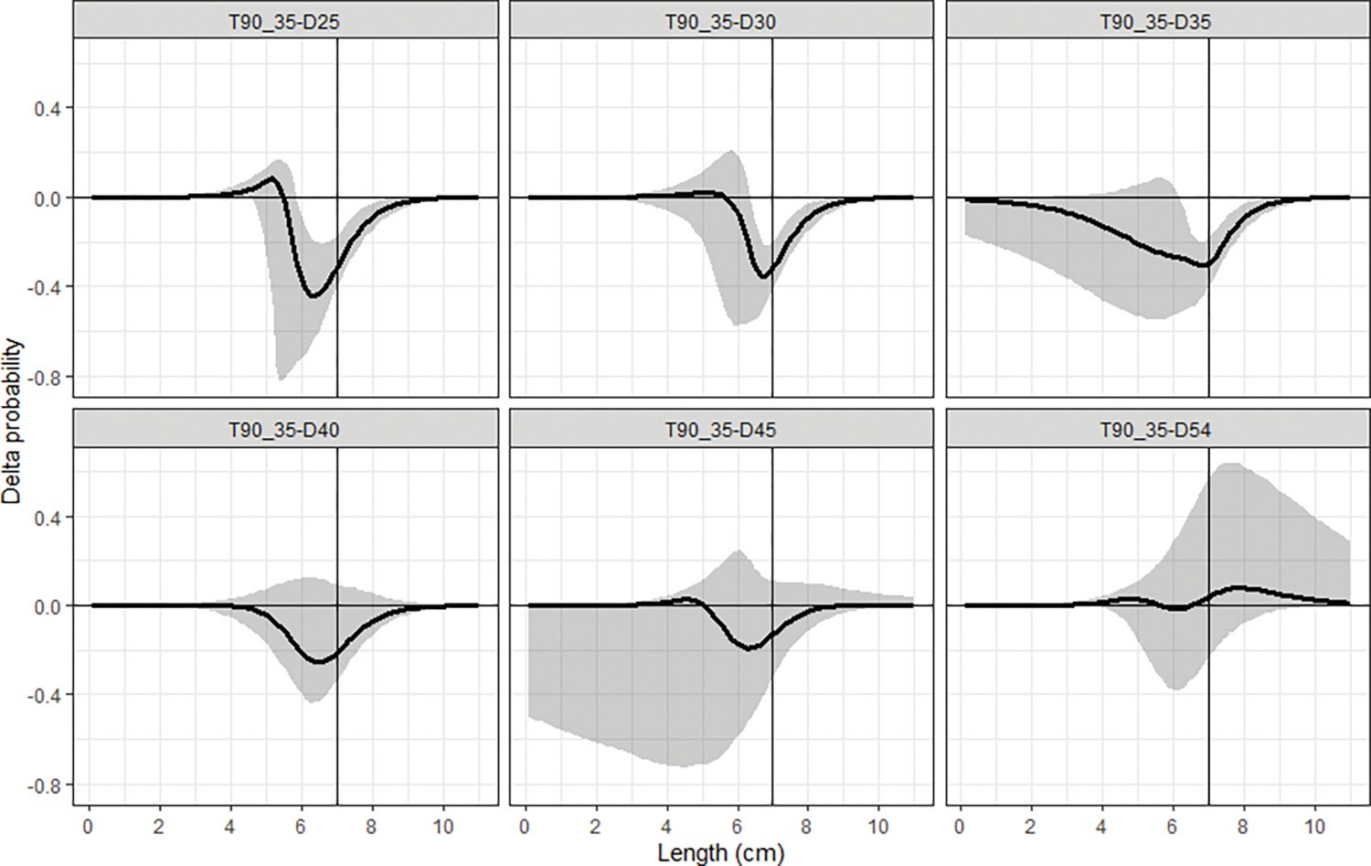
Delta selectivity of comparison between the T90_35 codend and the diamond-mesh codends tested by Yang and Herrmann [8]. The black curves represent the delta selectivity for comparison, and the shaded areas are the 95% confidence intervals. Vertical lines are the MCRS (minimum conservation reference size, 7.0 cm) of cocktail shrimp in the SCS.

We further compared the size selectivity of the T90_35 codend with the results of Yang and Herrmann [8]. The comparison demonstrated that the retention probability of the T90_35 codend was significantly lower than the three diamond-mesh codends with smaller mesh sizes for shrimp in the following length ranges: >5.9 cm than the D25 codend, >6.4 cm than the D30 codend and >6.2 cm than the D35 codend, respectively. Differences between the T90_35 codend and other three diamond-mesh codends with larger mesh sizes were not significant (Fig 5).

### Exploitation pattern indicators

In two different fishing population scenarios, the T0_30 codend had significantly higher retention fraction for cocktail shrimp with length both less and above the MCRS value. For instance, in the population of 2020 *nP-* and *nP+* was 80.37% and 99.70% for the T0_30 codend, while the related values dropped to 53.03% and 95.51% for the T90_30 codend. The values of *nP-* and *nP+* from the T90_30 codend were significantly higher than the T0_35 and T90_35 codend, in which no significant difference was found (Table 3). The discarded ratios (*dnRatio*) of the T0_30 codend was significantly higher than the T90_35 codend in the population scenario of 2019 and the T0_35 and T90_35 codend in fishing population scenario of 2020, respectively. (Table 3).

**Table 3:** Exploitation pattern indicators of cocktail shrimp obtained for the tested codends in two fishing population scenarios.

population	codend	*nP-*(%)	*nP+* (%)	*dnRatio* (%)
2019	T0_30	71.97 (59.00–79.69)	99.78 (99.27–99.97)	56.49 (46.87–65.27)
	T0_35	29.50 (23.25–35.86)	85.60 (79.43–91.52)	38.28 (29.80–46.91)
	T90_30	44.83 (35.46–54.08)	96.60 (94.35–98.07)	45.51 (36.91–53.91)
	T90_35	23.13 (14.55–31.76)	80.32 (73.69–88.17)	34.14 (23.34–45.29)
2020	T0_30	80.37 (69.47–85.37)	99.70 (99.02–99.95)	57.74 (50.90–63.48)
	T0_35	34.98 (29.17–40.75)	82.27 (75.47–89.14)	41.88 (34.94–47.72)
	T90_30	53.03 (43.63–60.41)	95.51 (92.72–97.40)	48.48 (41.54–54.00)
	T90_35	26.77 (17.15–35.25)	75.32 (67.87–83.98)	37.60 (27.61–45.40)

## Discussion

The results of our study have demonstrated that increasing the mesh size in diamond-mesh codend or substituting diamond-mesh codend with T90 codend would improve the size selectivity and exploitation pattern for cocktail shrimp in the SCS. The improvement can be further manifested by applying a T90 codend with a larger mesh size. Comparing with the starting point, the T0_30 codend, the benefits of increasing mesh size or/and applying T90 codend could be as: 1) significantly larger L50 values, 2) lower retention probability for undersized individuals of cocktail shrimp, and 3) more sustainable exploitation pattern, smaller values in *nP-* and *dnRatio*. Based on the comparison of selectivity, delta selectivity and exploitation pattern indicators, the T90_35 codend presented the best selective properties for cocktail shrimp in the studied areas.

Our study was the second research work to improve the size selectivity for cocktail shrimp in the SCS. The first one was conducted by Yang and Hermann [8]. They investigated the size selectivity of six diamond-mesh codends for cocktail shrimp, and found that the selective properties of the legal D25 was poor and the selectivity could not be sufficiently improved by simply increasing the mesh sizes in diamond-mesh codends. To further improve the size selectivity, it is suggested that T90 codends should be tested and documented [8]. Our study just follows this direction by testing and comparing the size selectivity and exploitation pattern between T0 and T90 codends. The results demonstrated that positive improvement in selective properties could be obtained when applying the T90 codends. Specifically, the T90_35 codend performed the best in selectivity parameters, delta selectivity and exploitation pattern indicators. Additionally, when comparing the size selectivity of the T90_35 codend with the results by Yang and Herrmann [8], it was showed that its L50 value was significantly larger than the diamond-mesh codends with mesh sizes less than 40 mm (D25, D30 and D35) while comparable to the diamond-mesh codends with larger mesh sizes (D40, D45 and D54) (Table 2). The benefits of applying the T90_35 codend could be further manifested by the delta selectivity with the results by Yang and Herrmann [8]. The implication of our study to the fisheries management is that the T90_35 codend could be a potential choice to substitute the present legal codend, D25, to mitigate the bycatch of undersized cocktail shrimp in shrimp trawl fisheries of the SCS.

Though our study was conducted in the SCS onboard a double-rigged trawler, it is relevant to compare our results with similar or related researches in a wider circumstance as cocktail shrimp is an economically important species widely distributed all across coastal waters in China. The earliest documentation of trawl codend selectivity for cocktail shrimp was conducted by Sun and Wang [38] in the late 1990s around the East China Sea. They tested three diamond-mesh codends, with mesh size as 40, 45 and 50 mm, respectively, and obtained L50 as 8.50, 8.60 and 8.83 cm, and SR as 1.39, 1.39 and 1.50 cm, respectively. Later, a similar experiment was carried out by Tang et al. [39] in the Yellow Sea. They used three mesh sizes of 30, 33 and 40 mm, and got L50 as 5.98, 6.01, and 6.32 cm, and SR as 1.44, 1.60 and 2.29 cm, respectively, for cocktail shrimp. More recently, a selectivity study was finished by Xu et al. [40] in the Bohai Sea applying five diamond-mesh codends, with mesh size as 15, 25, 35, 45 and 55 mm, respectively. Their results showed that L50 value was 2.46, 3.79, 4.41, 5.57, and 8.54 cm, while SR value was 1.19, 1.69, 1.95, 4.08 and 5.73 cm for cocktail shrimp. Although it is hard for us to directly compare our results with the previous studies, due to that they failed to provide CIs in their selectivity parameters, their results and our study share a similar trend as both L50 and SR increased as the mesh sizes of diamond-mesh codends increased.

Similar with the study by Yang and Herrmann [8], the results of our study demonstrated that a diamond-mesh codend with a mesh size a little bit larger than the legal MMS regulation (25 mm) presented poor selective properties for cocktail shrimp. For instance, the T0_30 codend in the present study had a small L50 value, high retention probability for undersized individuals and high discard ratios. Similar results were also reported by Tang et al. [39] and Xu et al. [40], in which small L50 values were obtained for diamond-mesh codend with a mesh size of 25 and 30 mm. The implication of all these results is that the national MMS regulation of 25 mm in codend should be revised for cocktail shrimp trawl fisheries all around the country. What we cannot explicitly explain is that why increasing the mesh size of T0 codend from 30 to 35 mm would improve size selectivity in our study but did not in study by Yang and Herrmann [8] which applying the codends with the same mesh sizes. One possible explanation might be the variability in the size selectivity of diamond-mesh codends due to their flexible characteristics.

T90 codends have been intensively tested and proved to be more selective than traditional diamond-mesh codends for many different species. The improvement of size selectivity in T90 codends can be due to improved opening of the meshes, from which the target species could easily escape [20,41–43]. The results of the present study are well in line with these previous studies. Escapement of shrimp species from the codend meshes was once recorded by our underwater video recordings, although we could not classify the escaped one into species level by the videos.

The results of the present study demonstrate that not only the MMS regulation should be revised, but also the formal MCRS or minimum landing size (MLS) needs to be made to improve the size selectivity and exploitation pattern for cocktail shrimp trawl fisheries in China. Unlike the size selectivity and curves, exploitation pattern indicators depend not only on the size structure of fishing population but also on the value of MLS. In our study, we chose a MCRS of 7.0 cm for the target species. A study by Yi and Liu [44] showed that the MLS should be 6.27 and 5.54 cm for female and male cocktail shrimp in the Yellow Sea. Research works are strictly needed to formulate an official MLS regulation for cocktail shrimp in nationwide or specific fishing areas. Because significant genetic differentiation of cocktail shrimp captured from different fishing areas has been revealed [2]. Irrespective of potential differences in fishing populations, it is highly relevant to mitigate bycatch of undersized individuals for cocktail shrimp trawl fisheries. As it has been reported that cocktail shrimp can live as long as four years [45].

Our results demonstrated that the bycatch of undersized cocktail shrimp could be significantly mitigated by applying the T90_35 codend. One possible drawback of using this codend, however, might by the loss of shrimp above the MCRS (*nP+*). Additionally, the selectivity curves became less shaper due to larger SR value. In order to further improve size selectivity and make it more recommendable to fishery management, more research works should be done. For instance, adding and shortening lastridge ropes on T0 or T90 codends should be considered and tested for cocktail shrimp fisheries in the SCS. Shortening lastridge ropes has been demonstrated as a simple and effective modification to improve size selectivity and exploitation by Sistiaga et al. [34,46]. This modification has great potential to be tested to further improve size selectivity in cocktail shrimp trawl fishery of the SCS.

## Conclusion

The results of our study have demonstrated that increasing the mesh size in diamond-mesh codend or substituting diamond-mesh codend with T90 codend would improve the size selectivity and exploitation pattern for cocktail shrimp in the SCS. Based on the comparison of selectivity, delta selectivity and exploitation pattern indicators, the T90_35 codend presented the best selective properties for cocktail shrimp in the studied areas.

## Supporting information

S1 File Data collected in the sea trials used for the analyses. The catch data consist of count data for cocktail shrimp in each experimental codend and cover for each size class.
(ZIP)

S2 File Images from GoPro HERO 4. One image was taken in natural condition and the other in red light.
(ZIP)
