# Supplementary figures and images for: Improving the size selectivity and exploitation pattern of cocktail shrimp (Trachypenaeus curvirostris) in shrimp trawl fishery of the South China Sea
Source: PLoS One. 2023 Dec 13;18(12):e0295776. doi: 10.1371/journal.pone.0295776 (PMC10718456; doi:10.1371/journal.pone.0295776)

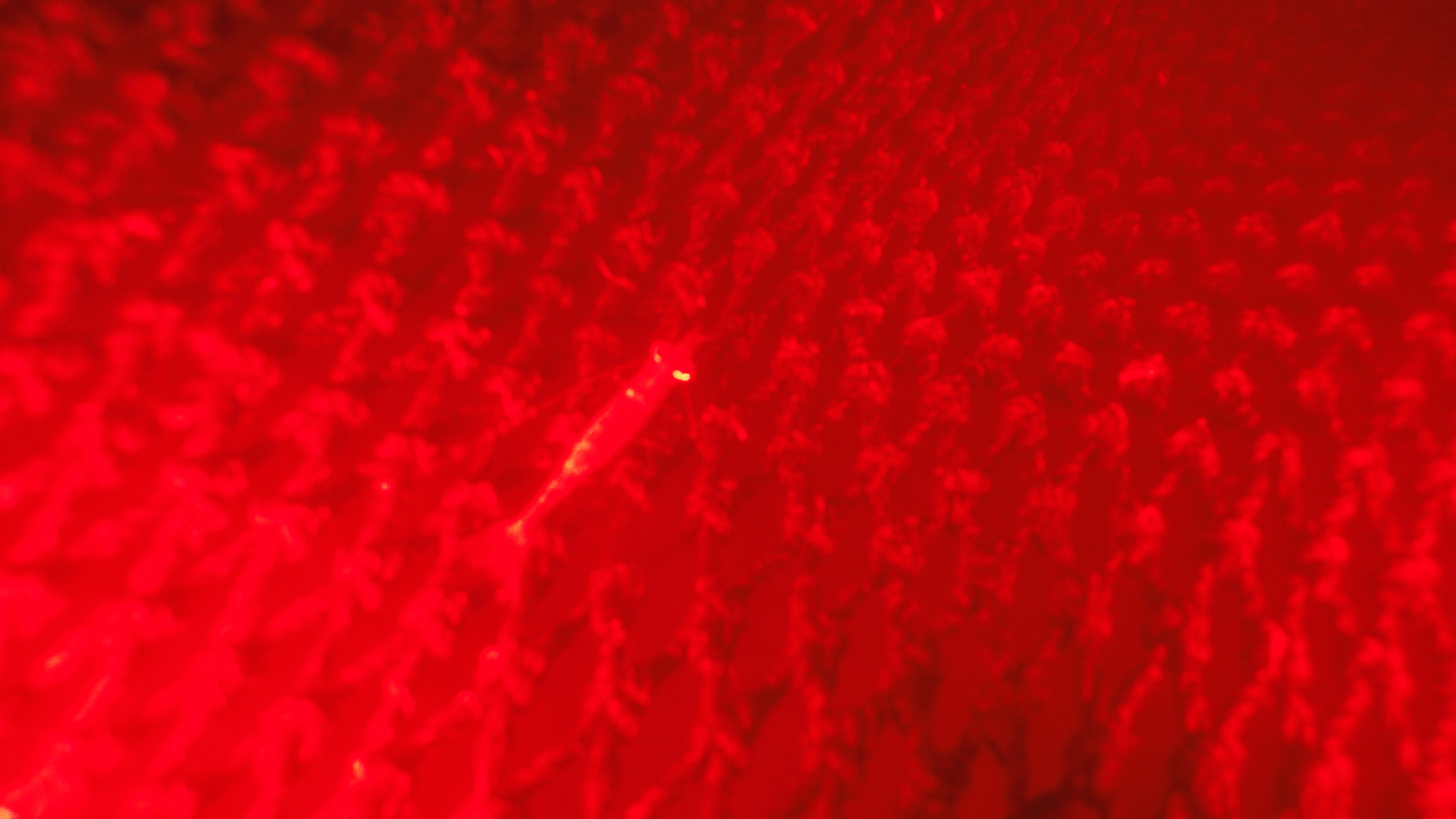

Supplement: S2 File — One image was taken in natural condition and the other in red light. (ZIP) [file pone.0295776.s002.zip › Image from GoPro HERO 4_red light.JPG]

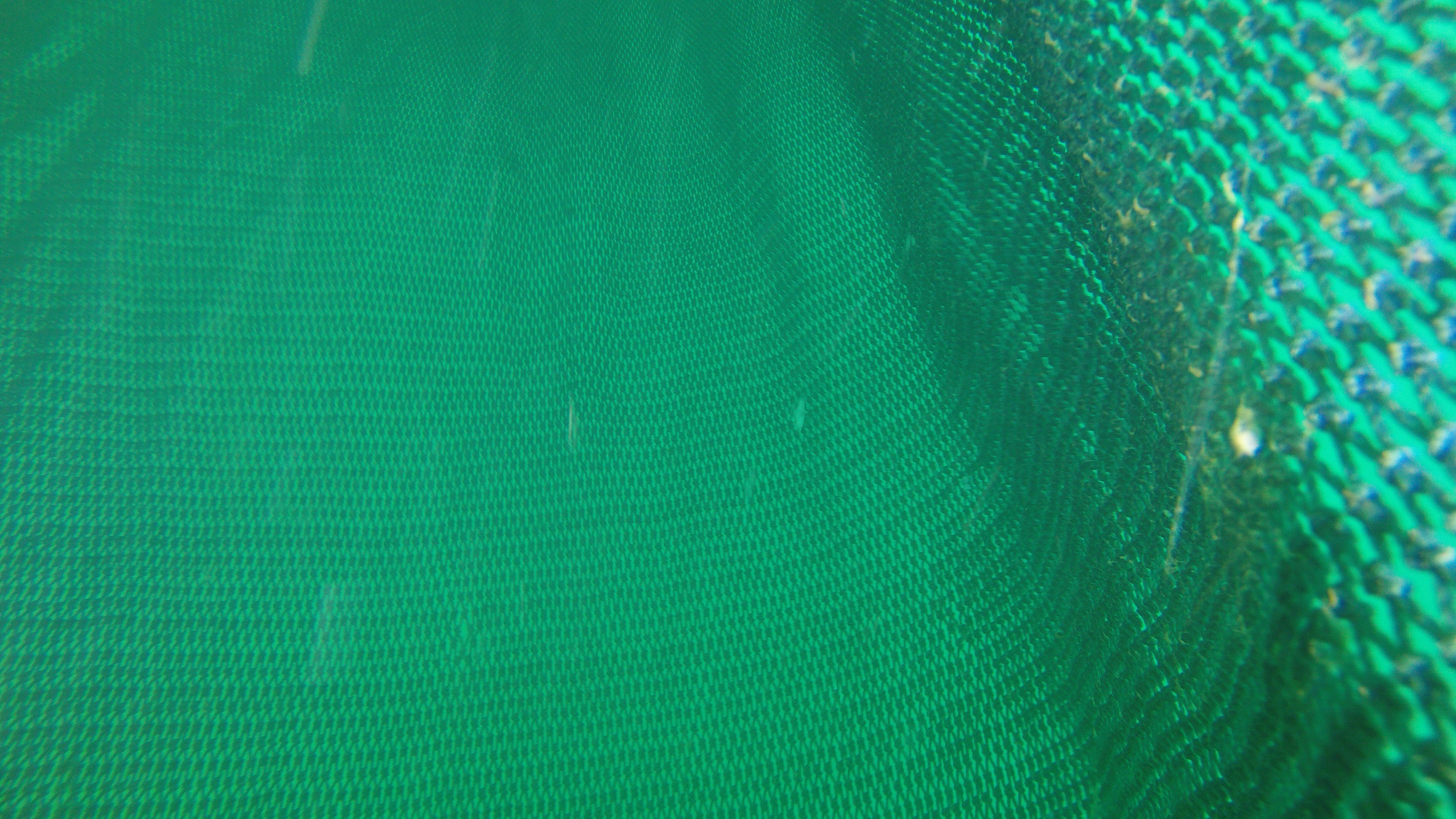

Supplement: S2 File — One image was taken in natural condition and the other in red light. (ZIP) [file pone.0295776.s002.zip › Image from GoPro HERO 4_natural condition.JPG]
